# Postoperative quality of life after minimally invasive repair of giant hiatal hernias

**DOI:** 10.1007/s10029-026-03804-6

**Published:** 2026-07-17

**Authors:** Flavio Tirelli, Laura Lorenzon, Cristina Galati, Francesca Chicchi, Gloria Santoro, Annamaria Agnes, Lorenzo Ferri, Roberto Persiani, Alberto Biondi, Domenico D’Ugo

**Affiliations:** 1 Fondazione Policlinico Universitario Agostino Gemelli IRCCS, Rome, Italy; 2 Università Cattolica del Sacro Cuore, Rome, Italy; 3 General Surgery Unit, Fondazione Policlinico Universitario “Agostino Gemelli”, Catholic University of the Sacred Heart, Largo Francesco Vito 1, 00168 Rome, Italy

**Keywords:** Hiatal hernia, Robotic surgery, Quality of life, Reflux disease, DeMeester symptom score

## Abstract

**Background:**

Type III and IV hiatal hernias require surgical repair to improve symptoms and prevent complications. Long-term patient-reported outcomes following minimally invasive repair, particularly with robotic-assisted techniques, remain incompletely described.

**Methods:**

This single-center retrospective cohort study evaluated consecutive patients who underwent elective hiatoplasty for type III or IV hiatal hernia between 2014 and 2024. Quality of life (QoL) was assessed using validated scores (GERD-HRQL, DeMeester symptom score) and a 5-point Likert satisfaction scale. The primary outcome of interest was postoperative QoL. Secondary outcomes included complications, recurrence, and associations with surgical technique (robotic vs. laparoscopic), mesh use, and fundoplication.

**Results:**

Eighty patients were selected from 152 treated during the study period. The cohort comprised 71.3% robotic-assisted and 22.5% laparoscopic procedures (6.2% converted). QoL was assessed at a median of 30 months’ follow-up. Postoperative median DeMeester symptom score was 1.0 (IQR 0.0–2.0), while median GERD-HRQL was 4.0 (IQR 0.0–8.0). The median satisfaction using the Likert scale was 5.0 (IQR 4.0–5.0). Major complications (Clavien-Dindo ≥ III) occurred in 5.0% of patients, with a 30-day readmission rate of 1.3%. Recurrence was documented in 21.2% (7 out of 33 patients with complete radiological follow-up data), with symptomatic recurrence in 6.1%. Higher BMI and symptomatic recurrence were significantly associated with lower quality-of-life scores (*p* < 0.05). No significant differences in postoperative QoL, complications, or recurrence were observed between robotic and laparoscopic approaches, with or without mesh reinforcement or fundoplication (*p* > 0.05).

**Conclusion:**

Minimally invasive hiatoplasty for type III and IV hiatal hernias achieves excellent long-term quality of life and high patient satisfaction independent of surgical approach, mesh use, or fundoplication. Preoperative BMI optimization, may enhance postoperative outcomes.

**Graphical abstract:**

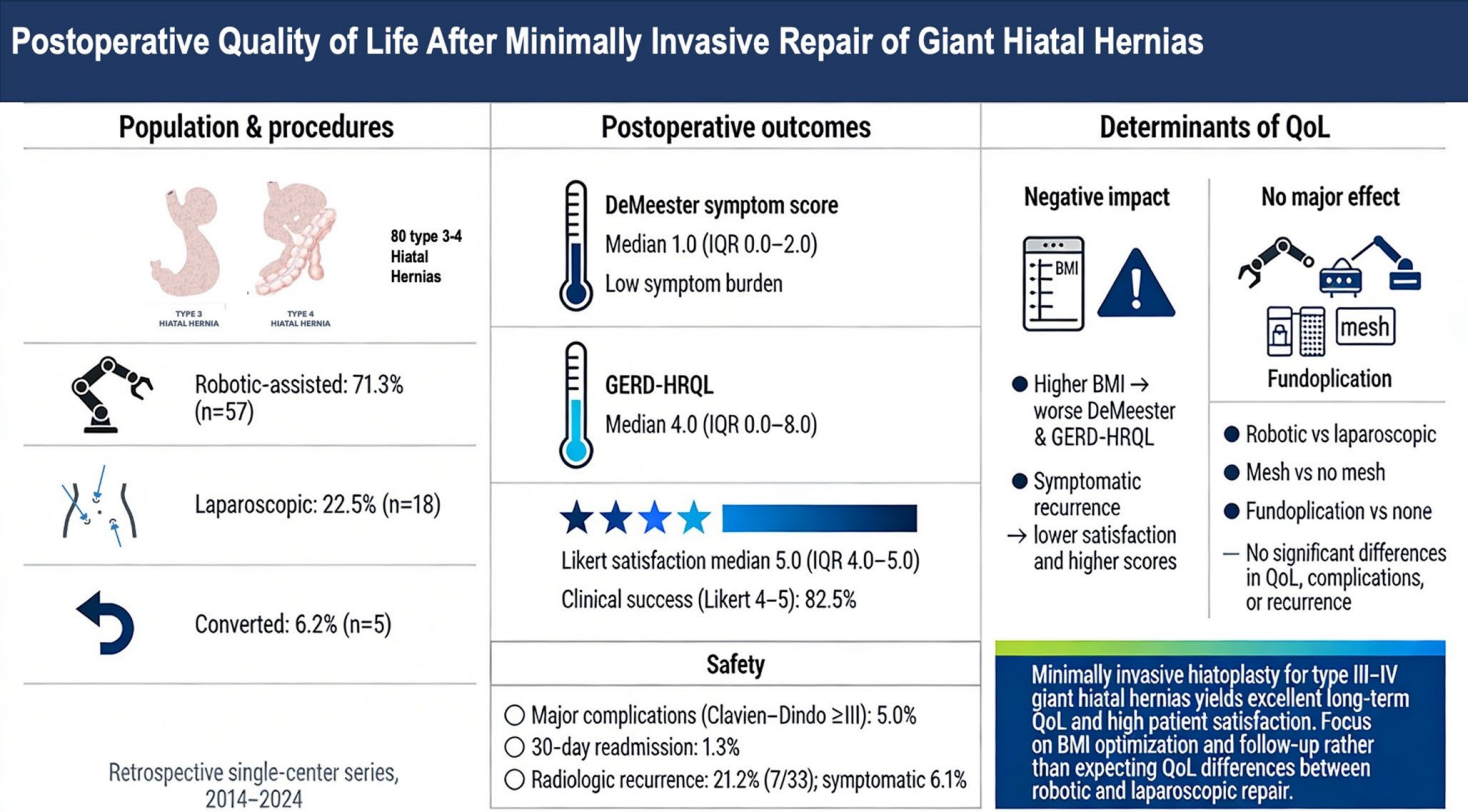

**Supplementary Information:**

The online version contains supplementary material available at 10.1007/s10029-026-03804-6.

## Introduction

A hiatal hernia is characterized by the herniation of abdominal viscera through the esophageal hiatus into the mediastinum. Pathogenesis relates to traumatic injury, increased intra-abdominal pressure, or weakening of the phreno-esophageal membrane [1]. According to the Hill classification, type I hernias involve displacement of the esophagogastric junction only; type II hernias feature gastric fundus herniation with the esophagogastric junction in normal position; type III hernias involve herniation of both the fundus and cardia; and type IV hernias encompass herniation of the entire stomach, often with additional abdominal organs [2].

Type I hiatal hernias are most common and typically asymptomatic with low complication risk. In contrast, large (type III and IV) hernias may cause symptoms proportional to the volume of herniated viscera, including gastroesophageal reflux, dysphagia, early satiety, postprandial vomiting, and cardiopulmonary compression [1]. Acute complications—bleeding, gastric incarceration, volvulus, and perforation—mandate surgical intervention. Surgical treatment remains the only curative option, involving reduction of herniated viscera and repair of the diaphragmatic defect, often with prosthetic mesh reinforcement, anti-reflux procedures (fundoplication), and/or gastropexy [3].

The primary goal of surgery is to improve quality of life (QoL) and prevent life-threatening complications. Patients undergoing elective repair often experience significant postoperative improvements in QoL [4]. Since the first laparoscopic hiatal hernia repairs in the early 1990s [5–13], minimally invasive surgery has become the standard of care, although early series reported high recurrence rates for giant hernias [14–15]. Nevertheless, large series have demonstrated sustained improvements in QoL irrespective of anatomic recurrence [4].

Following the first robotic-assisted Nissen fundoplication by Cadière [16], debate has emerged over whether robotic surgery offers advantages over conventional laparoscopy, including three-dimensional visualization and enhanced instrument articulation. Multiple studies have confirmed the safety and feasibility of robotic-assisted hiatal hernia repair [17–22].

Our institution has focused on minimally invasive treatment of upper gastrointestinal diseases over the past decade, with a dedicated robotic surgery program for benign conditions implemented since 2015–2016. Despite growing adoption of robotic techniques, patient-reported outcomes following repair of type III and IV hernias remain incompletely characterized, particularly in robotic-dominant programs. We aimed to evaluate postoperative quality of life after minimally invasive hiatoplasty for type III and IV hiatal hernias, examining associations with hernia type, surgical approach (robotic vs. laparoscopic), mesh use, and fundoplication.

## Materials and methods

### Study design and population

**Fig. 1:**
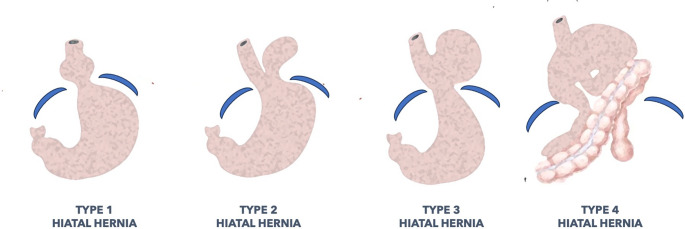
Hiatal hernia classification: schematic representation of hiatal hernias according to the Hill classification. Type III hernia is characterized by herniation of both the gastric fundus and the esophagogastric junction, whereas type IV hernia involves herniation of the entire stomach, often associated with other intra-abdominal organs

This single-center retrospective cohort study included all consecutive patients who underwent surgical hiatoplasty for hiatal hernia at Fondazione Policlinico Universitario A. Gemelli IRCCS between 2014 and 2024. Hernias were classified using the Hill system based on preoperative evaluation or intraoperative findings (Fig. 1). Patients who underwent elective hiatoplasty for type III or IV hiatal hernias were included. No size threshold was applied; all type III and IV hernias (irrespective of size) meeting the other criteria were included. Type I and II hernias were excluded as this study was specifically designed to address the outcomes of giant (type III and IV) hernias, which represent a distinct clinical entity with greater operative complexity and postoperative morbidity. Exclusion criteria comprised emergency surgery, loss to follow-up, or unwillingness to complete quality of life questionnaires.

Data collected included demographic variables (sex, age), clinical parameters (body mass index [BMI], Charlson Comorbidity Index, American Society of Anesthesiologists [ASA] score, associated diagnoses, previous abdominal surgery, smoking habits), operative details (mesh use, anti-reflux procedure or gastropexy, indocyanine green use, operative time, surgical approach), and postoperative outcomes (complications graded by Clavien-Dindo classification and functional results).

The research protocol was notified to the institutional review board. All procedures were performed in accordance with the 1964 Declaration of Helsinki and its later amendments.

### Preoperative assessment

All patients underwent esophagogastroduodenoscopy. Additional preoperative investigations included contrast swallow radiography, computed tomography, magnetic resonance imaging, high-resolution manometry, and 24-hour pH monitoring, depending on hernia type and symptoms. Imaging and functional studies were often performed before surgical referral by primary care physicians or gastroenterologists and completed as needed prior to surgery.

### Surgical technique

All procedures were performed by the same surgical team using laparoscopic, robotic, or open techniques, with preference for minimally invasive approaches. Robotic-assisted surgery for hiatal hernia repair became the preferred approach when the platform was available since 2015 (approximately once every 3 weeks); laparoscopy was used at other times. A dedicated robotic surgery program for benign upper gastrointestinal diseases (including hiatal hernia and achalasia) was formally implemented in 2017.

Laparoscopic surgery utilized a 4-trocar approach with energy device dissection. Robotic surgery was performed using the Da Vinci Xi platform (Intuitive Surgical, Inc., Sunnyvale, California, United States), as previously described [23]. Briefly, patients were positioned with legs abducted, the monitor placed at the right shoulder, the assistant standing between the legs, and in reverse Trendelenburg. Pneumoperitoneum was induced via Verres needle in the left hypochondrium. Four 8-mm robotic trocars were placed above the umbilicus: two in the right quadrant (one below the costal margin, one lateral to the rectus abdominis), one left of midline, and one in the left quadrant. A 12-mm assistant trocar was placed in the left iliac fossa. Following hernia sac dissection and reduction, suturing was performed by inverting the positions of the liver retractor and needle holder.

Hernia reduction was combined with hiatal repair via primary suturing, mesh reinforcement, or both. Suturing employed Mersilene (braided non-absorbable) or GORE-TEX interrupted sutures. Resorbable meshes included GORE BIO-A (glycolic acid/trimethylene carbonate synthetic U-shaped mesh, W. L. Gore & Associates, Inc., Newark, Delaware, United States) or Phasix Mesh (poly-4-hydroxybutyrate mesh, Becton, Dickinson and Company, Franklin Lakes, New Jersey, United States). Mesh type was selected according to availability in the hospital pharmacy. The repair approach (suturing and/or mesh reinforcement) and the use of gastropexy were determined intraoperatively based on the size of the hiatal defect: gastropexy was specifically performed in cases of large hiatal hernias where additional fixation was deemed necessary. The type of fundoplication (Nissen, Dor, or Toupet) was decided based on the presence of esophagitis on preoperative endoscopy and manometry evaluation when performed.

### Quality of life assessment

Functional outcomes were assessed using three validated instruments: the Gastroesophageal Reflux Disease Health-Related Quality of Life (GERD-HRQL) score, the DeMeester symptom score, and a 5-point Likert satisfaction scale.

The GERD-HRQL questionnaire was developed and validated to assess changes in typical GERD symptoms following medical or surgical treatment [24–25]. The total score is calculated by summing responses to 15 questions addressing heartburn, dysphagia, and regurgitation: questions 1–6 evaluate heartburn (heartburn sub-score), and questions 10–15 assess regurgitation (regurgitation sub-score). Each question is scored 0–5 (0 = no symptoms; 5 = symptoms severely affecting daily life), for a maximum total score of 75. The questionnaire accounts for proton pump inhibitor (PPI) use, considering that virtually all patients used PPIs preoperatively.

The DeMeester symptom score evaluates similar symptoms. Each symptom is scored 0–3, where 0 indicates the absence of the symptom and 3 indicates symptoms interfering with daily activities, with a maximum score of 9 [24–25]. The 5-point Likert satisfaction scale rates overall patient satisfaction (1 = “not satisfied”; 5 = “fully satisfied”). Questionnaires were administered via telephone interview during follow-up.

### Outcomes of interest

The primary outcome was postoperative quality of life assessed using validated GERD-HRQL and DeMeester questionnaires after type III and IV hernia repair, comparing results across the cohort and assessing variations by clinical characteristics. Secondary outcomes included differences by hernia type (III vs. IV) and surgical technique (robotic vs. laparoscopic) on recurrence, postoperative complications, Clavien-Dindo grade, length of hospital stay, and quality of life.

### Statistical analysis

An exploratory descriptive analysis characterized the cohort. Categorical variables were summarized using frequencies and percentages; numerical variables were summarized using means and standard deviations, medians, and interquartile ranges (IQRs). Categorical variables were analyzed using the chi-square test or Fisher’s exact test when appropriate. T-tests, Mann-Whitney U tests, and Kruskal-Wallis tests were used to compare continuous data based on normality. The DeMeester and GERD-HRQL questionnaires were dichotomized at their respective medians for group comparisons, after preliminary normality testing showing non-normal distribution (Shapiro-Wilk test for both DeMeester and GERD-HRQL *p* < 0.001). Therefore, dichotomization was implemented as a robust approach to mitigate the impact of extreme skewness and potential outliers in subsequent statistical models.

Parametric and nonparametric association tests estimated associations with outcomes including Clavien-Dindo score (0 vs. 1–5), mesh and fundoplication use, and surgical technique (laparoscopic vs. robotic). Variables with *p* ≤ 0.05 were deemed statistically significant. Missing data were handled by listwise deletion without imputation. All analyses were performed using R statistical software.

## Results

### Study population and baseline characteristics

**Fig. 2:**
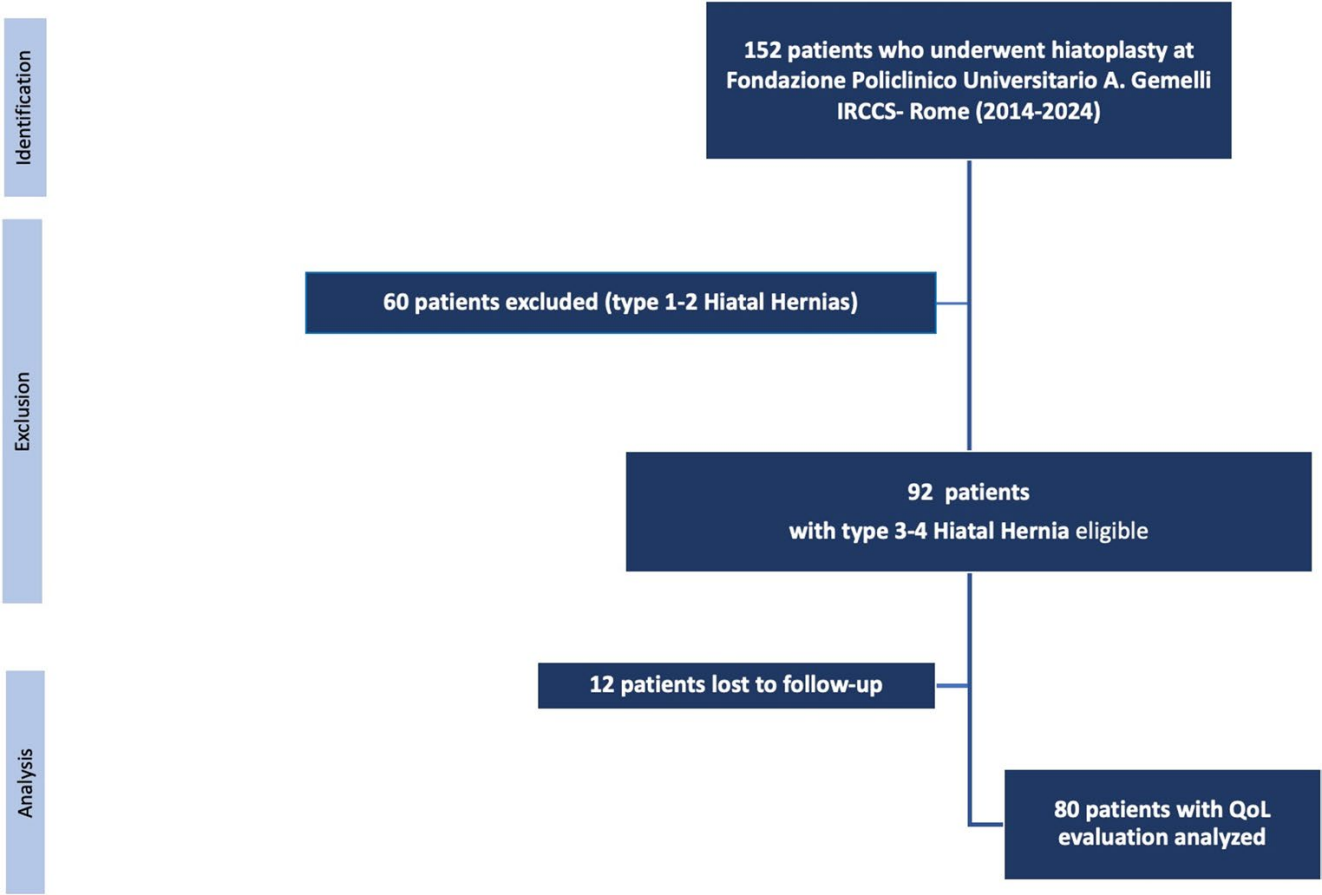
Study flow diagram

During the study period, 152 patients underwent surgical treatment for hiatal hernia. After excluding 60 patients with type I–II hernias, 92 patients were eligible. Twelve patients (13.0%) were lost to follow-up; thus, 80 patients with complete quality-of-life data were analyzed (Fig. 2). All questionnaires were administered at a mean follow-up of 31.5 months (median 30.0 months, IQR 13.8–47.3 months).

Table 1 presents baseline characteristics. Among 80 patients, females predominated (male-to-female ratio 0.43). Mean age was 66.7 years; mean preoperative BMI was 25.9. The majority had low comorbidity burden: only 7 patients had a Charlson index > 3, and 62.5% were ASA class 1–2. More than two-thirds (71.3%) had a GERD diagnosis with preoperative endoscopic findings. One patient was diagnosed with anemia secondary to Cameron’s ulcers.

**Table 1:** Clinical and surgical variables of patients who underwent surgery for type 3 and type 4 hiatal hernias

Sex	*n*	%
Female	56	70.0
Male	24	30.0
Age (years)		
Mean; Median (SD; IQR1-IQR3)	66.7; 67.2	(10.3; 59.7–74.8)
Smoking Habits	**n**	**%**
Non smoker	68	85.0
Smoker/Past smoker	12	15.0
Previous Abdominal Surgery	**n**	**%**
Yes	46	57.5
No	34	42.5
BMI		
Mean; Median (SD; IQR1-IQR3)	25.9; 26.1	(3.7; 23.7–28.5)
ASA Score	**n**	**%**
1–2	66	62.5
3–4	14	17.5
Charson Index	**n**	**%**
0	31	38.8
1	27	33.7
2	15	18.7
3	4	5.0
4	2	2.5
5	1	1.3
Associated Diagnosis	**n**	**%**
Yes (GERD)	57	71.3
No	23	28.7
Hiatal Hernia type	**n**	**%**
Type 3	57	71.3
Type 4	23	28.7
Surgical Approach		
Robotic Assisted	57	71.3
Laparoscopic	18	22.5
Converted	5	6.2
Operative time (minutes)		
Mean; Median (SD; IQR1-IQR3)	30.2; 31	(15.9; 19.0–43.0)
Mesh Use	**n**	**%**
Yes	44	55.0
No	36	45.0
Type of Mesh	**n**	**%**
Bio-A	40	90.9
Phasix	4	9.1
Fundoplication	**n**	**%**
Yes	39	48.8
No	41	51.2
Type of Fundoplication	**n**	**%**
Nissen	25	64.1
Dor	10	25.6
Toupet	4	10.3
Gastropexy	**n**	**%**
Yes	5	6.3
No	75	93.7
Post-Operative Length of Stay (days)		
Mean; Median (SD; IQR1-IQR3)	3.5; 3	(3.8; 2.0–4.0)
30-day Post-Operative Complications (Clavien-Dindo - C - Classification)	**n**	**%**
C0	72	90.0
C1-5	8	10.0
30-day Post-Operative Re-admission	**n**	**%**
Yes	1	1.3
No	79	98.7
Recurrence	**n**	**%**
Yes - symptoms	5	6.3
Yes - no symptoms (radiological recurrence)	2	2.5
No	26	32.5

*GERD* gastro-esophageal reflux disease

Preoperative imaging comprised X-ray swallow in 37 patients (46.2%), CT scans in 54 patients (67.5%), and abdominal MRI in 6 patients (7.5%). Functional studies included high-resolution manometry in 9 patients and pH monitoring in 10 patients.

Overall, 57 patients (71.3%) had type III hiatal hernia and 23 patients (28.7%) had type IV hernia. The surgical approach was robotic-assisted in 57 patients (71.3%) and laparoscopic in 18 patients (22.5%), with 5 conversions to open surgery (6.2%). Two conversions related to intraoperative brachy-esophagus findings; partial gastric reduction and gastropexy were achieved. Two conversions resulted from adhesions due to previous abdominal surgery, and one from intraoperative esophageal injury (see below). A progressive increase in total procedures was observed over the years, with a marked predominance of robotic interventions, consistent with our robotic-assisted surgery program for benign upper gastrointestinal disease (Supplementary Fig. 1).

### Operative details and perioperative outcomes

Intraoperative mesh placement was performed in 44 patients (55%); fundoplication in 39 patients (48.8%). Few cases were completed with gastropexy (6.3%). Mean postoperative stay was 3.5 days. Eight cases (10.0%) experienced 30-day complications. No deaths occurred. Four patients (5.0%) experienced major postoperative complications (Clavien-Dindo ≥C3): one required postoperative endoscopy for Forrest type 3 bleeding ulcer treated with glue and clips; another had intraoperative esophageal injury requiring conversion, ICU admission, second surgical procedure with cervicostomy, followed by restoration of continuity after approximately 2 months; one required ICU admission for 24 h for desaturation and was discharged with oxygen therapy; one developed pulmonary embolism. One 30-day readmission occurred for a fever treated with antibiotics. Radiological follow-up was obtained in 33 patients (41.2%); among these, 5 (15.5%) experienced symptomatic recurrence and 2 (6.1%) asymptomatic recurrence (overall recurrence rate 21.2%).

### Postoperative quality of life

**Fig. 3:**
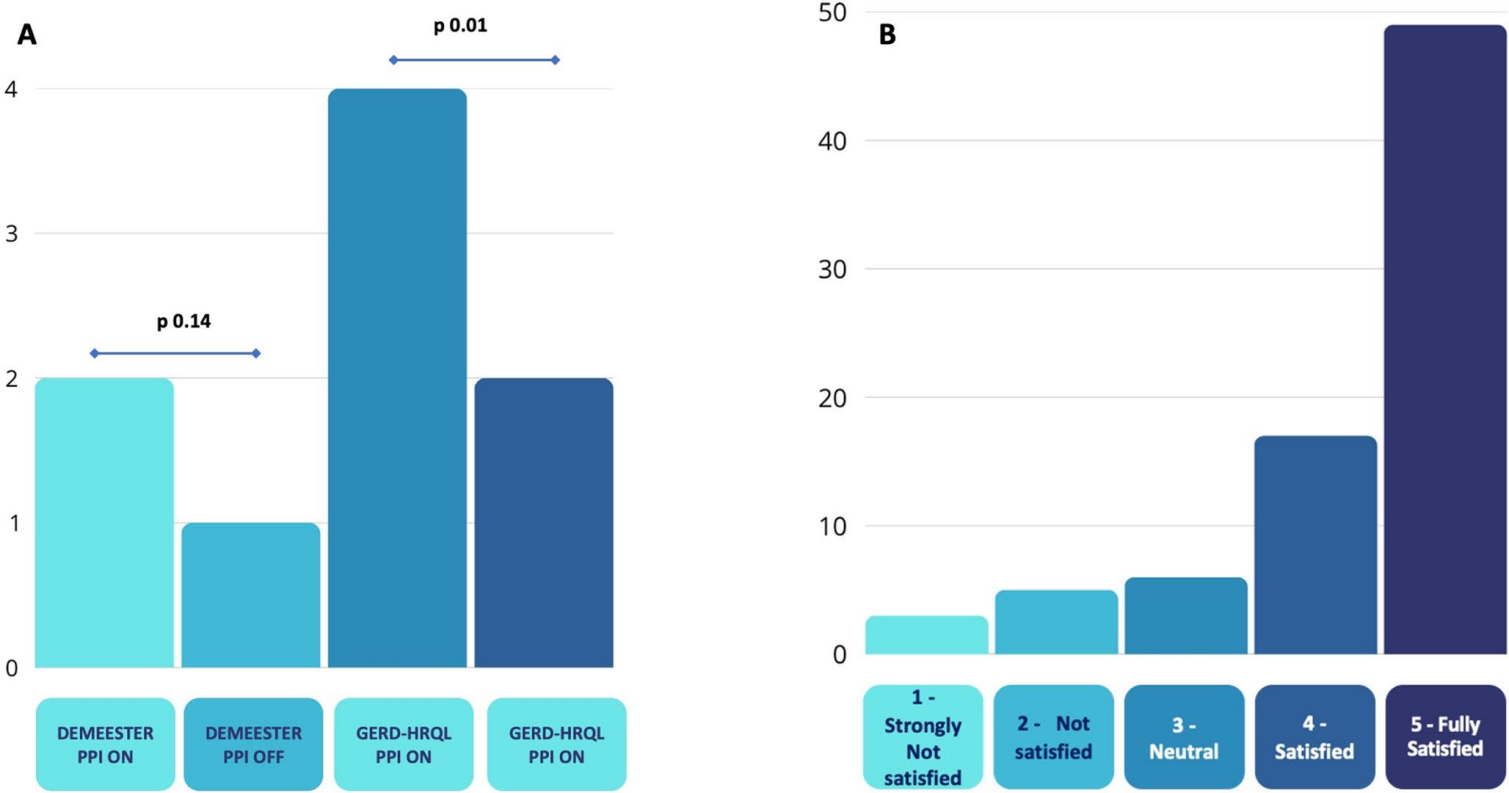
Postoperative quality of life. (**A**) Distribution of DeMeester and GERD-HRQL scores in patients on versus off PPIs at follow-up; (**B**) Distribution of overall patient satisfaction on the 5-point Likert scale

The QoL evaluation demonstrated excellent symptom control. Mean DeMeester symptom score was 1.7 (within non-pathologic range); mean total GERD-HRQL score was 6.7, with predominance of heartburn symptoms (mean heartburn domain 5.2 vs. mean regurgitation domain 3.6) (Table 2). Patients requiring postoperative PPI therapy (*n* = 43) had significantly higher median GERD-HRQL scores compared with PPI-free patients, whereas this difference was not observed for DeMeester symptom scores (PPI on vs. off: median GERD-HRQL 4 vs. 2, *p* = 0.01; median DeMeester 2 vs. 1, *p* = 0.14) (Fig. 3).

**Table 2:** Post-operative quality of life in patients who underwent surgery for type 3 and type 4 hiatal hernias

Demeester Score Overall		
Mean; Median (SD; IQR1-IQR3)	1.7; 1.0	(1.9; 0.0–2.0)
Demeester Score Heartburn Domain		
Mean; Median (SD; IQR1-IQR3)	0.7; 0.0	(0.9; 0.0–1.0)
Demeester Score Regurgitation Domain		
Mean; Median (SD; IQR1-IQR3)	0.7; 0.0	(0.9; 0.0–1.0)
Demeester Score Dysphagia Domain		
Mean; Median (SD; IQR1-IQR3)	0.3; 0.0	(0.7; 0.0–0.0)
GERD Health-Related Quality of Life (GERD-HRQL) Score Overall		
Mean; Median (SD; IQR1-IQR3)	6.7; 4.0	(11.5; 0.0–8.0)
GERD-HRQL Score Heartburn Domain		
Mean; Median (SD; IQR1-IQR3)	5.2; 6.0	(5.2; 1.0–11.0)
GERD-HRQL Score Regurgitation Domain		
Mean; Median (SD; IQR1-IQR3)	3.6; 3.6	(3.7; 1.0-6.2)
Likert Scale Score		
Mean; Median (SD; IQR1-IQR3)	4.3; 5.0	(1.1; 4.0–5.0)
Proton Pump Inhibitor Therapy	**n**	**%**
Yes	43	53.7
No	37	46.3

*GERD* gastro-esophageal reflux disease

Overall patient satisfaction was high: the mean Likert-scale assessment was 4.3 (Table 2). Clinical success was achieved in 66 patients reporting scores of 4 (21.2%) or 5 (61.2%); only 3 patients (3.7%) were strongly dissatisfied (Fig. 3).

Analysis of variables associated with patient-reported outcomes revealed that higher preoperative BMI and postoperative recurrence were significantly associated with worse quality of life, evidenced by higher median DeMeester and GERD-HRQL scores (Table 3). Likert satisfaction scores were significantly associated with recurrence rate (*p* = 0.007), with radiologically documented recurrence associated with lower overall satisfaction and higher median DeMeester and GERD-HRQL scores (*p* < 0.001) (Supplementary Table 1).

**Table 3:** Clinical and surgical variables correlated with quality of life according to Demeester and GERD-HRQL Scores

	Demeester ScoreBelow Median Values <143 patients	Demeester ScoreAbove Median Values ³237 patients	*p *value	GERD-HRQL ScoreBelow Median Values <235 patients	GERD-HRQL ScoreAbove Median Values ³345 patients	*p* value
Sex	n (%)	n (%)		n (%)	n (%)	
Female	30 (69.8)	26 (70.3)	1*	37 (68.5)	19 (73.1)	0.797*
Male	13 (30.2)	11 (29.7)		17 (31.5)	7 (26.9)	
Age (years)						
Median (IQR1-IQR3)	70.4 (60.0-75.3)	66.9 (59.4-71.0)	0.258^	70.3 (58.6-74.8)	67.3 (60.1-72.2)	0.333^^
Smoking Habits	n (%)	n (%)		n (%)	n (%)	
Non smoker	39 (90.7)	29 (78.4)	0.208*	47 (87.0)	21 (80.8)	0.512*
Smoker/Past smoker	4 (9.3)	8 (21.6)		7 (13.0)	5 (19.2)	
Previous Abdominal Surgery	n (%)	n (%)		n (%)	n (%)	
Yes	26 (60.5)	20 (54.1)	0.652*	31 (57.4)	15 (57.7)	1*
No	17 (39.5)	17 (45.9)		23 (42.6)	11 (42.3)	
BMI						
Mean (SD)	25.1 (3.3)	26.9 (3.8)	0.029^^	25.2 (3.3)	27.5 (4.0)	0.009^^
ASA Score	n (%)	n (%)		n (%)	n (%)	
1	4 (9.3)	2 (5.4)	0.315*	4 (7.4)	2 (7.7)	1*
2	34 (79.1)	26 (70.3)		40 (74.1)	20 (76.9)	
3	5 (11.6)	9 (24.3)		10 (18.5)	4 (15.4)	
4	0 (0.0)	0 (0.0)		0 (0.0)	0 (0.0)	
Associated Diagnosis	n (%)	n (%)		n (%)	n (%)	
Yes (GERD)	29 (67.4)	28 (75.7)	0.534*	37 (68.0)	20 (76.9)	0.625*
No	14 (32.6)	9 (24.3)		17 (31.5)	6 (23.1)	
Hiatal Hernia type	n (%)	n (%)		n (%)	n (%)	
Type 3	30 (69.8)	27 (73.0)	0.808*	37 (66.7)	20 (76.9)	0.599*
Type 4	13 (30.2)	10 (27.0)		17 (31.5)	6 (23.1)	
Surgical Approach	n (%)	n (%)		n (%)	n (%)	
Robotic Assisted						
Yes	30 (69.8)	31 (83.8)	0.19*	42 (77.8)	19 (73.1)	0.78*
No	13 (30.2)	6 (16.2)		12 (22,2)	7 (26.9)	
Laparoscopic						
Yes	13 (30.2)	6 (16.2)	0.19*	12 (22.2)	67 (26.9)	0.19*
No	30 (69,8)	31 (83.8)		42 (77.8)	19 (73.1)	
Converted						
Yes	2 (4.7)	3 (8.1)	0.658*	3 (5.6)	2(7.7)	0.658*
No	41 (95.3)	34 (91.9)		51 (94.4)	24 (92.3)	
Mesh Use	n (%)	n (%)		n (%)	n (%)	
BIO-A	18 (41.9)	22 (59.5)	0.266*	27 (50.0)	13 (50.0)	1*
Phasix	3 (7.0)	1 (2.7)		3 (5.6)	1 (3.8)	
None	22 (51.2)	14 (37.8)		24.4 (44.4)	12 (46.2)	
Fundoplication	n (%)	n (%)		n (%)	n (%)	
Yes	20 (46.5)	19 (51.4)	0.823*	29 (53.7)	10 (38.5)	0.238*
No	23 (53.5)	18 (48.6)		25 (46.3)	16 (61.5)	
Type of Fundoplication	n (%)	n (%)		n (%)	n (%)	
Nissen	15 (34.9)	10 (27.0)	0.123*	18 (33.3)	7 (26.9)	0.7*
Dor	2 (4.7)	8 (21.6)		8 (14.8)	2 (7.7)	
Toupet	3 (7.0)	1 (2.7)		3 (5.6)	1 (3.8)	
None	23 (53.5)	18 (48.6)		25 (46.3)	16 (61.5)	
Gastropexy	n (%)	n (%)		n (%)	n (%)	
Yes	4 (9.3)	1 (2.7)	0.366*	4 (7.4)	1 (3.8)	1*
No	39 (90.7)	36 (97.3)		50 (92.6)	25 (96.2)	
30-day Post-Operative Complications (Clavien-Dindo - C - Classification)	n (%)	n (%)		n (%)	n (%)	
C0	38 (88.4)	34 (91.9)	0.603*	48 (88.9)	24 (92.3)	0.635*
C1-C5	5 (11.6)	3 (8.1)		6 (11.1)	2 (7.7)	
30-day Post-Operative Re-admission	n (%)	n (%)		n (%)	n (%)	
Yes	1 (2.3)	0 (0.0)	1*	1 (1.9)	0 (0.0)	1*
No	42 (97.7)	37 (100.0)		53 (98.1)	26 (100.0)	
Recurrence	n (%)	n (%)		n (%)	n (%)	
Yes - symptoms	0 (0.0)	5 (13.5)	0.043*	0 (0.0)	5 (19.2)	0.008*
Yes - no symptoms (radiological recurrence)	0 (0.0)	2 (5.4)		1 (1.9)	1 (3.8)	
No	13 (30.2)	13 (35.1)		18 (33.3)	8 (30.8)	
Missing	30 (69.8)	17 (45.9)		35 (64.8)	12 (46.2)	

*GERD* gastro-esophageal reflux disease

*Chi Square test; ^ Mann-Whitney U test; ^^T test

### Subgroup analysis by Hernia type

Among patients with type III hiatal hernia, no significant correlation was identified between evaluated parameters and DeMeester symptom scores; however, recurrences significantly affected GERD-HRQL results (*p* = 0.01) and Likert-scale satisfaction (*p* = 0.038) (Supplementary Table 2). In patients with type IV hernia, postoperative complications significantly affected Likert-scale satisfaction (*p* = 0.003), whereas a borderline significant association was observed between DeMeester symptom score and surgical approach (*p* = 0.046) (Supplementary Table 3). Patients with higher BMI exhibited higher GERD scores, reflecting more severe symptomatology. A detailed overview of the associations between clinical–surgical variables and postoperative DeMeester and GERD-HRQL scores is reported in Table 4.

**Table 4:** Clinical and surgical variables associated with different hiatal hernia types

	Hiatal HerniaType 357 patients	Hiatal HerniaType 423 patients	p value
Sex	n (%)	n (%)	
Female	42 (73.7)	14 (60.9)	0.289*
Male	15 (26.3)	9 (39.1)	
Age (years)			
Median (IQR1-IQR3)	67.8 (58.3 -73.8)	71.0 (64.5 -75.6)	0.200^
Smoking Habits	n (%)	n (%)	
Non smoker	49 (86.0)	19 (82.6)	0.736*
Smoker/Past smoker	8 (14.0)	4 (17.4)	
Previous Abdominal Surgery	n (%)	n (%)	
Yes	33 (57.9)	13 (56.5)	1*
No	24 (42.1)	10 (43.5)	
BMI			
Mean (SD)	26.2 (3.4)	25.3 (4.2)	0.321^^
ASA Score	n (%)	n (%)	
1	5 (8.8)	1 (4.3)	0.76*
2	43 (75.4)	17 (73.9)	
3	9 (15.8)	5 (21.7)	
4	0 (0.0)	0 (0.0)	
Associated Diagnosis	n (%)	n (%)	
Yes (GERD)	44 (77.2)	13 (56.5)	0.15*
No	13 (22.8)	10 (43.5)	
Surgical Approach	n (%)	n (%)	
Robotic Assisted			
Yes	43 (75.4)	18 (78.3)	1*
No	14 (24.6)	5 (21.7)	
Laparoscopic			
Yes	14 (24.6)	5 (21.7)	1*
No	43 (75.4)	18 (78.3)	
Converted			
Yes	3 (5.3)	2(8.7)	0.622*
No	54 (94.7)	21 (91.3)	
Fundoplication	n (%)	n (%)	
Yes	30 (52.6)	9 (39.1)	0.328*
No	27 (47.4)	14 (60.9)	
Type of Fundoplication	n (%)	n (%)	
Nissen	19 (33.3)	6 (26.1)	0.585*
Dor	7 (12.3)	3 (13.0)	
Toupet	19 (33.3)	6 (26.1)	
None	27 (47.4)	14 (60.9)	
Gastropexy	n (%)	n (%)	
Yes	1 (1.8)	4 (17.4)	0.022*
No	56 (98.2)	19 (82.6)	
Surgical Approach (intention-to-treat)	n (%)	n (%)	
Robotic Assisted	43 (75.4)	18 (78.3)	1*
Laparoscopic	14 (24.6)	5 (21.7)	
30-day Post-Operative Complications (Clavien Dindo - C - Classification)	n (%)	n (%)	
C0	55 (96.5)	17 (73.9)	0.006*
C1-C5	2 (3.5)	6 (26.1)	
30-day post-operative Re-admission	n (%)	n (%)	
Yes	0 (0.0)	1 (4.3)	0.288*
No	57 (100.0)	22 (95.7)	
Recurrence	n (%)	n (%)	
Yes - symptoms	17 (29.8)	9 (39.1)	1
Yes - no symptoms (radiological recurrence)	4 (7.0)	1 (4.3)	
No	1 (1.8)	1 (4.3)	
Missing	35 (61.4)	12 (52.2)	
Likert Scale Score	n (%)	n (%)	
1	3 (5.3)	0 (0.0)	0.755
2	3 (5.3)	2 (8.7)	
3	5 (8.8)	1 (4.3)	
4	13 (22.8)	4 (17.4)	
5	33 (57.9)	16 (69.6)	
Demeester Score			
Median (IQR1-IQR3)	1.0 (0.0 -2.0)	1.0 (0.0 -2.0)	0.445^
GERD-HRQL Score			
Median (IQR1-IQR3)	4.0 (1.0 -9.0)	3.0 (0.0-6.0)	0.291^

*GERD* gastro-esophageal reflux disease

*Chi Square test; ^ Mann-Whitney U test; ^^ T test

### Subgroup analysis by surgical approach

Comparing laparoscopic and robotic-assisted surgery, operative time was significantly longer for robotic procedures (*p* < 0.001) (Supplementary Table 4). However, perioperative outcomes, including postoperative complications, postoperative length of stay, recurrence rate, and long-term quality of life, were similar between groups (DeMeester, GERD-HRQL, and Likert-scale scores; all *p* > 0.05).

No significant differences were observed comparing patients treated with mesh reinforcement versus suturing alone, or patients undergoing fundoplication versus hiatoplasty without fundoplication, regarding postoperative complications, postoperative stay, recurrence rate, and long-term quality of life (all *p* > 0.05) (Supplementary Table 5). Similarly, no significant difference was documented when comparing QoL outcomes with the type of fundoplication (Dor and Toupet vs. Nissen, all *p* > 0.05) (Supplementary Table 6).

## Discussion

This study demonstrates excellent long-term quality of life and high patient satisfaction following minimally invasive repair of type III and IV hiatal hernias, with over 80% of patients achieving DeMeester symptom scores ≤ 2 at a median 30-month follow-up. Our findings contribute to the growing literature on patient-reported outcomes after giant hernia repair, with particular focus on outcomes from a predominantly robotic-assisted surgical program.

Hiatal hernia affects approximately 15% of the global population; however, most patients present with type I hernias, which are typically asymptomatic [2]. Large hernias produce symptoms including gastroesophageal reflux, dysphagia, early satiety, postprandial vomiting, and cardiopulmonary compression. Surgical treatment remains the only curative option, often incorporating prosthetic mesh with or without anti-reflux procedures or gastropexy [1].

Our results align with prior large series demonstrating sustained quality-of-life improvement following giant hiatal hernia repair [4]. The very low median DeMeester overall and domain scores, combined with high median Likert satisfaction (5.0, IQR 4–5), support the narrative of excellent long-term symptom control. These outcomes are consistent with systematic reviews of quality of life after giant hiatus hernia repair, which have shown significant improvements in GERD-related symptoms and patient satisfaction scores.

A significant correlation was observed between higher BMI and increased likelihood of symptomatic recurrence, with these patients presenting higher DeMeester and GERD-HRQL scores. BMI represents a major risk factor for both hiatal hernia development and surgical treatment success. These findings underscore the importance of addressing obesity and related metabolic conditions before surgical intervention. A multidisciplinary preoperative assessment is advisable to achieve a BMI < 25 prior to surgery and maintain a healthy lifestyle thereafter. Tailored approaches should include nutritional counseling with personalized diet plans, regular physical activity, and smoking cessation—all of which are critical for sustaining long-term postoperative benefits.

The importance of BMI as a risk factor for esophageal disorders is now universally recognized. According to the available literature [26], BMI should be considered when selecting a surgical approach, although our series did not demonstrate significant QoL differences between robotic and laparoscopic approaches.

One study investigated whether esophageal motility disorders influenced postoperative outcomes, finding comparable results between populations; thus, motility disorders are not contraindications to hiatoplasty [27].

When evaluating various surgical factors—mesh use, robotic versus laparoscopic approach, and fundoplication addition—our results showed largely comparable outcomes for functional outcomes, complications, hospital stay, and readmission rates. No statistically significant differences emerged between patients who were operated on using laparoscopic or robotic techniques, nor between those who underwent mesh placement or fundoplication and those who did not.

These findings are consistent with recent literature on robotic versus laparoscopic hiatal hernia repair. Multiple studies have demonstrated comparable perioperative and functional outcomes between approaches, though robotic surgery is associated with longer operative time and higher costs. A multicenter study comparing different meshes (Bio-A and Phasix) showed no clear superiority, although Phasix may be associated with greater tissue incorporation due to slower resorption [28].

The lack of robotic superiority has been demonstrated by several studies [29–32]; however, robotic surgery is associated with longer operative time and higher costs [32, 33]. Conversely, one study including 8,019 patients demonstrated comparable one-month follow-up results between surgical techniques, whereas one-year follow-up showed reduced symptomatic recurrence rate with robotic surgery [34].

These findings may reflect our decade-long experience, which enabled adequate standardization of surgical technique across approaches. In our predominantly robotic program, both techniques achieved excellent quality-of-life outcomes, consistent with the literature suggesting that surgeon experience and proper technique selection based on patient characteristics are more important than inherent approach superiority.

Analysis of surgical variables revealed that gastropexy was the only factor significantly associated with postoperative outcomes. Among patients with Clavien-Dindo grade 0, only 3.7% underwent gastropexy, whereas among those with higher grades, 36.4% underwent gastropexy.

Another study evaluated the efficacy of laparoscopic gastropexy without fundoplication using the GERD-HRQL questionnaire, finding non-inferior functional outcomes but an approximately 30% recurrence rate and a complication rate [35]. This may reflect that patients requiring gastropexy typically present more advanced or complex hiatal hernias necessitating this additional surgical step. In our series, gastropexy was performed selectively in complex cases, which may explain the association with complications rather than gastropexy itself being causative.

Patients not taking PPIs exhibited significantly higher DeMeester questionnaire scores, whereas no significant differences were observed in GERD-HRQL scores between PPI users and non-users. This underscores the concerns regarding long-term PPI use, associated with conditions including enteric infections, microscopic colitis, kidney disease, and hypomagnesemia [36]. Nevertheless, prolonged PPI therapy remains recommended in selected cases [37]. The relationship between persistent symptoms, PPI use, and QoL scores requires careful interpretation: patients with residual symptoms may be more likely to continue PPI therapy, and the questionnaires themselves account for PPI use in symptom assessment. Of note, PPIs are routinely prescribed for 4 weeks after surgery [38], and then suspended according to the patient’s symptoms and clinical evaluation.

Strengths of this study include the use of validated patient-reported outcome measures (GERD-HRQL, DeMeester symptom score, Likert satisfaction scale), relatively long median follow-up (30 months), and focus on a well-defined population (type III and IV hernias only) from a high-volume center with an established robotic surgery program. The predominantly robotic approach (71.3%) provides contemporary data relevant to current trends in surgical practice.

Limitations include the retrospective single-center design, which limits generalizability. The absence of preoperative QoL scores for all patients prevents calculation of absolute symptom change, though postoperative scores alone demonstrate a good symptom control within the non-pathologic range. Furthermore, radiological follow-up study was missing in a significant proportion of cases, as many patients were referred for surgery from different Italian districts, asymptomatic patients without clinical concerns were not routinely referred for imaging, and the study relied primarily on clinical evaluation and QoL questionnaires at follow-up. While this represents a limitation, the postoperative scores achieved are within the non-pathologic range, supporting the clinical benefit of surgery. Telephone follow-up may introduce recall bias. High rates of missing data for radiologic recurrence (58.7%) limit firm conclusions about the relationship between anatomic recurrence and quality of life, though available data show significant associations. Dichotomization of continuous QoL scores, while appropriate given the absence of validated cutoffs, reduces statistical power. Finally, the non-randomized nature precludes definitive conclusions about comparative effectiveness of surgical approaches, mesh use, or fundoplication, though the lack of significant differences across approaches is reassuring. Indeed, while the use of fundoplication is low compared to other large datasets, others documented that most patients with large HH do not have reflux as the main presenting symptom, advocating for a more tailored approach [39–40].

## Conclusion

Minimally invasive hiatoplasty for type III and IV hiatal hernias achieves excellent long-term quality of life and high patient satisfaction at a median 30-month follow-up. Postoperative quality of life is not significantly influenced by hernia type, surgical approach (robotic vs. laparoscopic), mesh reinforcement, or use of fundoplication, with all approaches demonstrating acceptable complication rates and low recurrence rates. Higher preoperative BMI and symptomatic recurrence are significant predictors of worse postoperative quality of life. Comprehensive preoperative evaluation, including nutritional and multidisciplinary assessment, is essential to ensure optimal surgical outcomes and long-term patient well-being. Proper patient selection, weight optimization, and lifestyle modification should be considered integral components of the therapeutic strategy for managing large hiatal hernias.

## Supplementary Information

Below is the link to the electronic supplementary material.

Supplementary Material 1

## Data Availability

Research data supporting this publication are available from the corresponding author on reasonable request.
