# Supplementary material for: Postoperative quality of life after minimally invasive repair of giant hiatal hernias
Source: Hernia. 2026 Jul 17;30(1):295. doi: 10.1007/s10029-026-03804-6 (PMC13379444; doi:10.1007/s10029-026-03804-6)
Supplement: Supplementary file 1 — Supplementary Material 1 [file 10029_2026_3804_MOESM1_ESM.docx]

**Postoperative Quality of Life After Minimally Invasive Repair of Giant Hiatal Hernias**

**Running Title.** Hiatal hernia and quality of life

**Article type.** Original Article

**Supplementary Materials - Index**

| **Type - Supplementary Figure** |  |
| --- | --- |
| Supplementary Figure 1. Surgical approach over the years | *page 2* |
| **Type - Supplementary Tables** |  |
| Supplementary Table 1. Patients' overall satisfaction according to 5-point Likert scale and clinical-surgical variables in type 3 and type 4 hiatal hernias | *page 3* |
| Supplementary Table 2. Patients' quality of life and post-operative satisfaction and clinical surgical variables in type 3 hiatal hernia | *page 6* |
| Supplementary Table 3. Patients' quality of life, post-operative satisfaction, and clinical surgical variables in type 4 hiatal hernia | *page 8* |
| Supplementary Table 4. Clinical and surgical variables according to surgical approach (intention-to-treat) in type 3 and type 4 hiatal hernias | *page 10* |
| Supplementary Table 5. Post-operative complications and quality of life according to mesh use and fundoplication in type 3 and type 4 hiatal hernias. | *page 12* |
| Supplementary Table 6: Type of Fundoplication and Quality of Life | *page 13* |

**Supplementary Figure 1. Surgical approach over the years**

**A**


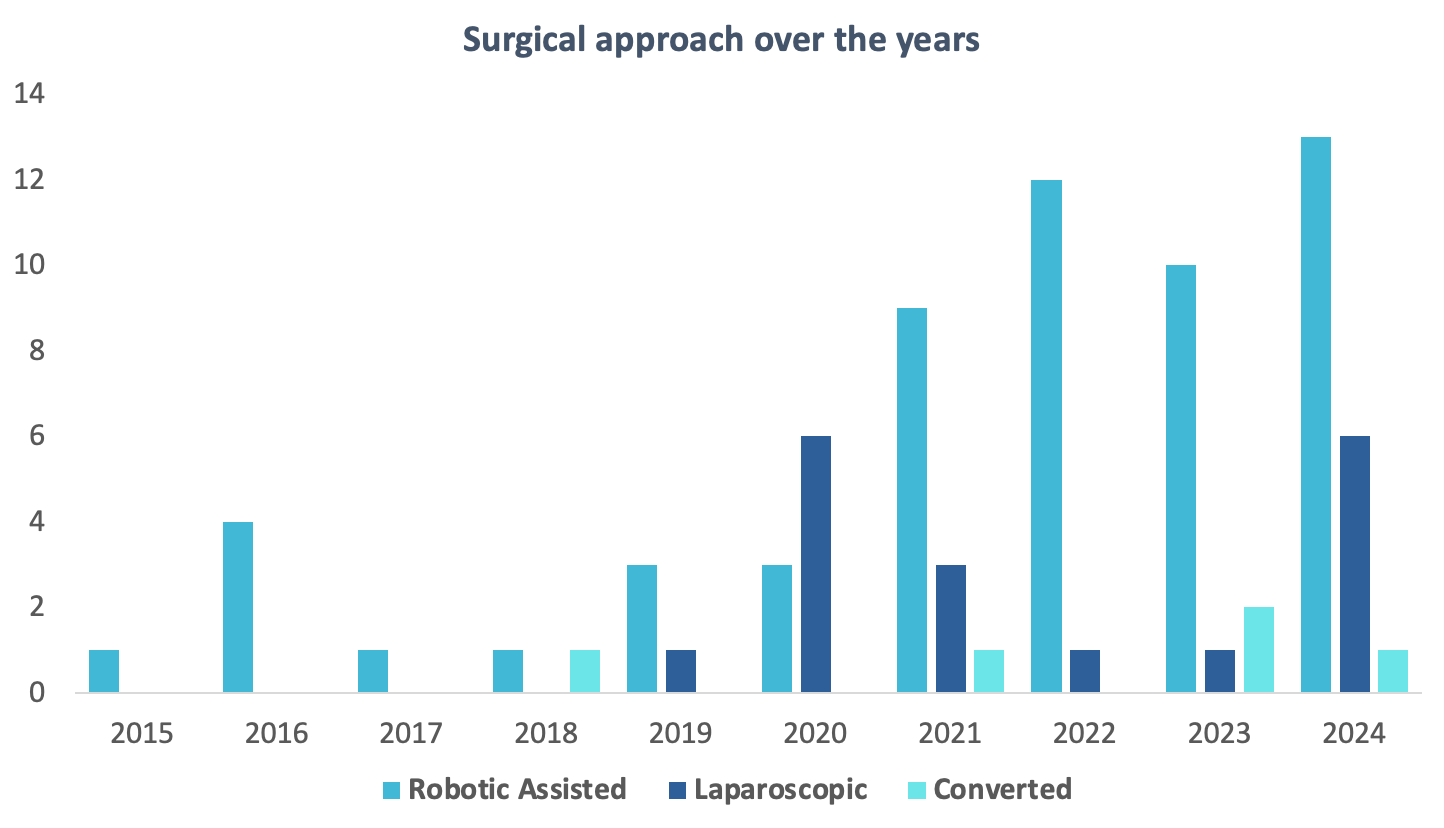


**B**


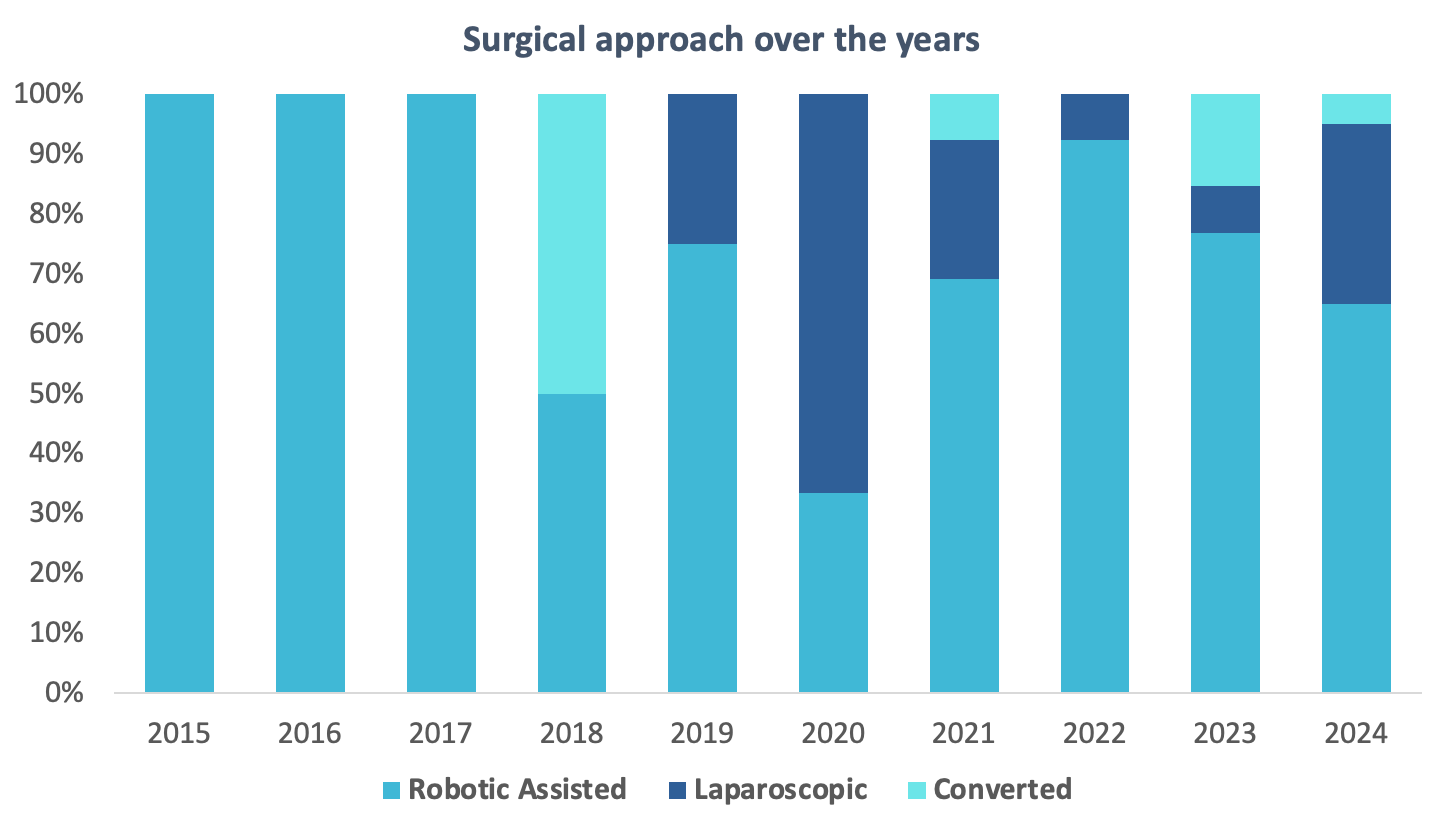


**Supplementary Figure 1. A.** Number of surgical procedures according to the approach over the years; **B.** Percentages of robotic-assisted. laparoscopic. and converted procedures over the years.

**Supplementary Table 1. Patients' overall satisfaction according to 5-point Likert scale and clinical-surgical variables in type 3 and type 4 hiatal hernias**

|  | **Likert 1**  **3 patients n (%)** | **Likert 2**  **5 patients n (%)** | **Likert 3**  **6 patients n (%)** | **Likert 4**  **17 patients**  **n (%)** | **Likert 5**  **49 patients**  **n (%)** | **p value** |
| --- | --- | --- | --- | --- | --- | --- |
| **Sex** |  |  |  |  |  |  |
| Female | 2 (66.7) | 3 (60.0) | 4 (66.7) | 14 (82.4) | 33 (67.3) | 0.764* |
| Male | 1 (33.3) | 2 (40.0) | 2 (33.3) | 3 (17.6) | 16 (32.7) |  |
| **Age (years)** |  |  |  |  |  |  |
| Median (IQR1-IQR3) | 67.1 (53.9-69.0) | 63.7 (59.4-70.3) | 67.4 (65.4-76.5) | 70.3 (60.1-75.1) | 68.1 (59.6 - 74.8) | 0.865^ |
| **BMI** |  |  |  |  |  |  |
| Median (IQR1-IQR3) | 26.6 (26.4 - 27.8) | 25.7 (25.4 -27.4) | 27.2 (25.2 -30.0) | 25.8 (24.8 (29.1) | 24.6 (22.6 -28.0) | 0.104^ |
| **Smoking Habits** |  |  |  |  |  |  |
| Non Smoker | 3 (100.0) | 4 (80.0) | 3 (50.0) | 16 (94.1) | 42 (85.7) | 0.142* |
| Smoker/Past smoker | 0 (0.0) | 1 (20.0) | 3 (50.0) | 1 (5.9) | 7 (14.3) |  |
| **Previous Abdominal Surgery** | |  |  |  |  |  |
| Yes | 1 (33.3) | 3 (60.0) | 2 (33.3) | 8 (47.1) | 32 (65.3) | 0.384* |
| No | 2 (66.7) | 2 (40.0) | 4 (66.7) | 9 (52.9) | 17 (34.7) |  |
| **ASA Score** |  |  |  |  |  |  |
| ASA 1 | 1 (33.3) | 0 (0.0) | 1 (16.7) | 0 (0.0) | 4 (8.2) | 0.235* |
| ASA 2 | 2 (66.7) | 3 (60.0) | 3 (50.0) | 15 (88.2) | 37 (75.5) |  |
| ASA 3 | 0 (0.0) | 2 (40.0) | 2 (33.3) | 2 (11.8) | 8 (16.3) |  |
| ASA 4 | 0 (0.0) | 0 (0.0) | 0 (0.0) | 0 (0.0) | 0 (0.0) |  |
| **Charlson Index** |  |  |  |  |  |  |
| 0 | 2 (66.7) | 1 (20.0) | 3 (50.0) | 3 (17.6) | 22 (44.9) | 0.6* |
| 1 | 1 (33.3) | 4 (80.0) | 1 (16.7) | 7 (41.2) | 14 (28.6) |  |
| 2 | 0 (0.0) | 0 (0.0) | 2 (33.3) | 4 (23.5) | 9 (18.4) |  |
| 3 | 0 (0.0) | 0 (0.0) | 0 (0.0) | 2 (11.8) | 2 (4.1) |  |
| 4 | 0 (0.0) | 0 (0.0) | 0 (0.0) | 1 (5.9) | 1 (2.0) |  |
| 5 | 0 (0.0) | 0 (0.0) | 0 (0.0) | 0 (0.0) | 1 (2.0) |  |
| **Associated Diagnosis** | |  |  |  |  |  |
| Yes (GERD) | 2 (66.7) | 5 (100.0) | 4 (66.7) | 10 (58.8) | 36 (73.4) | 0.665* |
| No | 1 (33.3) | 0 (0.0) | 2 (33.3) | 7 (41.2) | 13 (26.5) |  |
| **Hiatal Hernia Type** | |  |  |  |  |  |
| Type 3 | 3 (100.0) | 3 (60.0) | 5 (83.3) | 13 (76.5) | 33 (67.3) | 0.755* |
| Type 4 | 0 (0.0) | 2 (40.0) | 1 (16.7) | 4 (23.5) | 16 (32.7) |  |
| **Surgical Approach** | |  |  |  |  |  |
| Robotic Assisted | |  |  |  |  |  |
| Yes | 2 (66.7) | 4 (80.0) | 5 (83.3) | 12 (70.6) | 38 (77.6) | 0.938* |
| No | 1 (33.3) | 1 (20.0) | 1 (16.7) | 5 (29.4) | 11 (22.4) |  |
| Laparoscopic |  |  |  |  |  |  |
| Yes | 1 (33.3) | 1 (20.0) | 1 (16.7) | 5 (29.4) | 11 (22.4) | 0.938* |
| No | 2 (66.7) | 4 (80.0) | 5 (83.3) | 12 (70.6) | 38 (77.6) |  |
| Converted |  |  |  |  |  |  |
| Yes | 0 (0.0) | 1 (20.0) | 1 (16.7) | 1 (5.9) | 2 (4.1) | 0.254* |
| No | 3 (100.0) | 4 (80.0) | 5 (83.3) | 16 (94.1) | 47 (95.9) |  |
| **Mesh Use** |  |  |  |  |  |  |
| Yes | 1 (33.3) | 2 (40.0) | 3 (50.0) | 9 (52.9) | 29 (59.2) | 0.822* |
| No | 2 (66.7) | 3 (60.0) | 3 (50.0) | 8 (47.1) | 20 (40.8) |  |
| **Type of Mesh** |  |  |  |  |  |  |
| Bio-A | 1 (33.3) | 1 (20.0) | 3 (50.0) | 8 (47.1) | 27 (55.1) | 0.679* |
| Phasix | 0 (0.0) | 1 (20.0) | 0 (0.0) | 1 (5.9) | 2 (4.1) |  |
| None | 2 (66.7) | 3 (60.0) | 3 (50.0) | 8 (47.1) | 20 (40.8) |  |
| **Fundoplication** |  |  |  |  |  |  |
| Yes | 2 (66.7) | 3 (60.0) | 2 (33.3) | 6 (35.3) | 26 (53.1) | 0.627* |
| No | 1 (33.3) | 2 (40.0) | 4 (66.7) | 11 (64.7) | 23 (46.9) |  |
| **Type of Fundoplication** | |  |  |  |  |  |
| Nissen | 2 (66.7) | 3 (60.0) | 2 (33.3) | 3 (17.6) | 15 (30.6) | 0.863* |
| Dor | 0 (0.0) | 0 (0.0) | 0 (0.0) | 2 (11.8) | 8 (16.3) |  |
| Toupet | 0 (0.0) | 0 (0.0) | 0 (0.0) | 1 (5.9) | 3 (6.1) |  |
| None | 1 (33.3) | 2 (40.0) | 4 (66.7) | 11 (64.7) | 23 (46.9) |  |
| **Gastropexy** |  |  |  |  |  |  |
| Yes | 0 (0.0) | 0 (0.0) | 0 (0.0) | 2 (11.8) | 3 (6.1) | 0.85* |
| No | 3 (100.0) | 5 (100.0) | 6 (100.0) | 15 (88.2) | 46 (93.9) |  |
| **30-day Post-Operative Complications (Clavien-Dindo - C - Classification)** | | | | |  |  |
| C0 | 3 (100.0) | 5 (100.0) | 6 (100.0) | 13 (76.5) | 45 (91.8) | 0.425* |
| C1-5 | 0 (0.0) | 0 (0.0) | 0 (0.0) | 4 (23.5) | 4 (8.2) |  |
| **30-day Post-Operative Complications (Clavien-Dindo - C - Classification)** | | | | | | |
| C0 | 3 (100.0) | 5 (100.0) | 6 (100.0) | 13 (76.5) | 45 (91.8) | 0.719* |
| C1 | 0 (0.0) | 0 (0.0) | 0 (0.0) | 0 (0.0) | 1 (2.0) |  |
| C2 | 0 (0.0) | 0 (0.0) | 0 (0.0) | 2 (11.8) | 1 (2.0) |  |
| C3 | 0 (0.0) | 0 (0.0) | 0 (0.0) | 1 (5.9) | 1 (2.0) |  |
| C4 | 0 (0.0) | 0 (0.0) | 0 (0.0) | 1 (5.9) | 1 (2.0) |  |
| C5 | 0 (0.0) | 0 (0.0) | 0 (0.0) | 0 (0.0) | 0 (0.0) |  |
| **Post-Operative Length of Stay (days)** | | |  |  |  |  |
| Mean (SD) | 2.7 (0.6) | 2.8 (0.4) | 3.2 (1.5) | 5.4 (7.7) | 3.0 (1.3) | 0.247* |
| **30-day Post-Operative Re-admission** | | |  |  |  |  |
| Yes | 0 (0.0) | 0 (0.0) | 0 (0.0) | 0 (0.0) | 1 (2.0) | 1* |
| No | 3 (100.0) | 5 (100.0) | 6 (100.0) | 17 (100.0) | 48 (98.0) |  |
| Missing | 0 (0.0) | 0 (0.0) | 0 (0.0) | 0 (0.0) | 0 (0.0) |  |
| **Recurrence** |  |  |  |  |  |  |
| Yes - symptoms | 1 (33.3) | 2 (40.0) | 1 (16.7) | 1 (5.9) | 0 (0.0) | **0.007*** |
| Yes - no symptoms (radiological recurrence) | 0 (0.0) | 0 (0.0) | 1 (16.7) | 0 (0.0) | 1 (2.0) |  |
| No | 0 (0.0) | 1 (20.0) | 2 (33.3) | 7 (41.2) | 16 (32.7) |  |
| Missing | 2 (66.7) | 2 (40.0) | 2 (33.3) | 9 (52.9) | 32 (65.3) |  |
| **Demeester Score Overall** | |  |  |  |  |  |
| Median (IQR1-IQR3) | 9.0 (5.0-0.0) | 5.0 (3.0-5.0) | 4.0 (3.3-4.8) | 2.0 (1.0-2.0) | 0.0 (0.0-1.0) | **<0.001^** |
| **GERD Health-Related Quality of Life (GERD-HRQL) Score Overall** | | | | |  |  |
| Median (IQR1-IQR3) | 56.0 (32.0-62.5) | 19.0 (9.0-35.0) | 9.5 (6.5-17.8) | 7.0 (4.0-9.0) | 1.0 (1.0-3.0) | **<0.001^** |

GERD: gastro-esophageal reflux disease; *Chi-square Test; ^ Kruskal-Wallis test

**Supplementary Table 2. Patients' quality of life and post-operative satisfaction and clinical surgical variables in type 3 hiatal hernia**

|  | **Demeester Score**  **Below Median Values < 1** | **Demeester Score**  **Above Median Values ≥ 2** | **p value** | **GERD-HRQL Score**  **Below Median Values <5** | | **GERD-HRQL Score**  **Above Median Values ≥ 6** | **p value** | **Likert 1** | **Likert 2** | | **Likert 3** | **Likert 4** | | | **Likert 5** | **p value** |
| --- | --- | --- | --- | --- | --- | --- | --- | --- | --- | --- | --- | --- | --- | --- | --- | --- |
|  | **30 patients n (%)** | **27 patients n (%)** |  | **37 patients n (%)** | | **20 patients n (%)** |  | **3 patients n (%)** | **3 patients n (%)** | | **5 patients n (%)** | **13 patients n (%)** | | | **33 patients n (%)** |  |
| **Sex** |  |  |  |  |  | |  |  |  |  | | |  |  | |  |
| Female | 23 (76.7) | 19 (70.4) | 0.764* | 27 (73.0) | | 15 (75.0) | 1* | 2 (66.7) | 2 (66.7) | | 3 (60.0) | 11 (84.6) | | | 24 (72.7) | 0.743* |
| Male | 7 (23.3) | 8 (29.6) |  | 10 (27.0) | | 5 (25.0) |  | 1 (33.3) | 1 (33.3) | | 2 (40.0) | 2 (15.4) | | | 9 (27.3) |  |
| **Age (years)** |  |  |  |  | |  |  |  |  | |  |  | | |  |  |
| Mean (SD) | 67.2 (9.5) | 64.4 (11.6) | 0.321^ | 67.3 (10.0) | | 63.4 (11.4) | 0.185^ | 59.6 (16.4) | 59.2 (4.6) | | 67.0 (17.6) | 68.5 (8.5) | | | 65.9 (10.0) | 0.5^^ |
| **Smoking Habits** | |  |  |  | |  |  |  |  | |  |  | | |  |  |
| Non smoker | 27 (90.0) | 22 (81.5) | 0.457* | 33 (89.2) | | 16 (80.0) | 0.432* | 3 (100.0) | 2 (66.7) | | 3 (60.0) | 12 (92.3) | | | 29 (87.9) | 0.273* |
| Smoker/Past smoker | 3 (10.0) | 5 (18.5) |  | 4 (10.8) | | 4 (20.0) |  | 0 (0.0) | 1 (33.3) | | 2 (40.0) | 1 (7.7) | | | 4 (12.1) |  |
| **Previous Abdominal Surgery** | | |  |  | |  |  |  |  | |  |  | | |  |  |
| Yes | 20 (66.7) | 13 (48.1) | 0.187* | 23 (62.2) | | 10 (50.0) | 0.411* | 1 (33.3) | 1 (33.3) | | 2 (40.0) | 6 (46.2) | | | 23 (69.7) | 0.287* |
| No | 10 (33.3) | 14 (51.9) |  | 14 (37.8) | | 10 (50.0) |  | 2 (66.7) | 2 (66.7) | | 3 (60.0) | 7 (53.8) | | | 10 (30.3) |  |
| **BMI** |  |  |  |  | |  |  |  |  | |  |  | | |  |  |
| Mean (SD) | 25.6 (3.3) | 26.9 (3.5) | 0.154^ | 25.8 (3.2) | | 27.1 (3.7) | 0.184^ | 27.3 (1.6) | 25.3 (2.3) | | 26.8 (2.7) | 27.4 (3.5) | | | 25.7 (3.6) | 0.5958^^ |
| **ASA Score** |  |  |  |  | |  |  |  |  | |  |  | | |  |  |
| ASA 1 | 3 (10.0) | 2 (7.4) | 0.535* | 3 (8.1) | | 2 (10.0) | 0.721* | 1 (33.3) | 0 (0.0) | | 1 (20.0) | 0 (0.0) | | | 3 (9.1) | 0.34* |
| ASA 2 | 24 (80.0) | 19 (70.4) |  | 27 (73.0) | | 16 (80.0) |  | 2 (66.7) | 3 (100.0) | | 2 (40.0) | 11 (84.6) | | | 25 (75.8) |  |
| ASA 3 | 3 (10.0) | 6 (22.2) |  | 7 (18.9) | | 2 (10.0) |  | 0 (0.0) | 0 (0.0) | | 2 (40.0) | 2 (15.4) | | | 5 (15.2) |  |
| ASA 4 | 0 (0.0) | 0 (0.0) |  | 0 (0.0) | | 0 (0.0) |  | 0 (0.0) | 0 (0.0) | | 0 (0.0) | 0 (0.0) | | | 0 (0.0) |  |
| **Associated Diagnosis** | | |  |  | |  |  |  |  | |  |  | | |  |  |
| Yes (GERD) | 23 (76.7) | 21 (77.8) | 1* | 28 (75.7) | | 16 (80.0) | 0.839* | 2 (66.7) | 3 (100.0) | | 3 (60.0) | 9 (69.2) | | | 27 (82.2) | 0.732* |
| No | 7 (23.3) | 6 (22.2) |  | 9 (24.3) | | 4 (20.0) |  | 1 (33.3) | 0 (0.0) | | 2 (40.0) | 4 (30.8) | | | 6 (18.2) |  |
| **Surgical Approach** | |  |  |  | |  |  |  |  | |  |  | | |  |  |
| Robotic Assisted |  |  |  |  | |  |  |  |  | |  |  | | |  |  |
| Yes | 22 (73.3) | 21 (77.8) | 0.765* | 29 (78.4) | | 14 (70.0) | 0.53* | 2 (66.7) | 2 (66.7) | | 4 (80.0) | 9 (69.2) | | | 26 (78.8) | 0.877* |
| No | 8 (26.7) | 6 (22.2) |  | 8 (21.6) | | 6 (30.0) |  | 1 (33.3) | 1 (33.3) | | 1 (20.0) | 4 (30.8) | | | 7 (21.2) |  |
| Laparoscopic |  |  |  |  | |  |  |  |  | |  |  | | |  |  |
| Yes | 8 (26.7) | 6 (22.2) | 0.765* | 8 (21.6) | | 6 (30.0) | 0.53* | 1 (33.3) | 1 (33.3) | | 1 (20.0) | 4 (30.8) | | | 7 (21.2) | 0.877* |
| No | 22 (73.3) | 21 (77.8) |  | 29 (78.4) | | 14 (70.0) |  | 2 (66.7) | 2 (66.7) | | 4 (80.0) | 9 (69.2) | | | 26 (78.8) |  |
| Converted |  |  |  |  | |  |  |  |  | |  |  | | |  |  |
| Yes | 2 (6.7) | 1 (3.7) | 1* | 2 (5.4) | | 1 (5.0) | 1* | 0 (0.0) | 0 (0.0) | | 1 (20.0) | 0 (0.0) | | | 2 (6.1) | 0.579* |
| No | 28 (93.3) | 26 (96.3) |  | 35 (94.6) | | 19 (95.0) |  | 3 (100.0) | 3 (100.0) | | 4 (80.0) | 13 (100.0) | | | 31 (93.9) |  |
| **Type of Mesh** |  |  |  |  | |  |  |  |  | |  |  | | |  |  |
| BIO-A | 11 (36.7) | 16 (59.3) | 0.119* | 17 (45.9) | | 10 (50.0) | 0.802* | 1 (33.3) | 1 (33.3) | | 2 (40.0) | 7 (53.8) | | | 16 (48.5) | 0.918* |
| Phasix | 2 (6.7) | 0 (0.0) |  | 2 (5.4) | | 0 (0.0) |  | 0 (0.0) | 0 (0.0) | | 0 (0.0) | 1 (7.7) | | | 1 (3.0) |  |
| None | 17 (56.7) | 11 (40.7) |  | 18 (48.6) | | 10 (50.0) |  | 2 (66.7) | 2 (66.7) | | 3 (60.0) | 5 (38.5) | | | 16 (48.5) |  |
| **Fundoplication** | | |  |  | |  |  |  |  | |  |  | | |  |  |
| Yes | 16 (53.3) | 14 (51.9) | 1* | 21 (56.8) | | 9 (45.0) | 0.42* | 2 (66.7) | 3 (100.0) | | 2 (40.0) | 5 (38.5) | | | 18 (54.5) | 0.42* |
| No | 14 (46.7) | 13 (48.1) |  | 16 (43.2) | | 11 (55.0) |  | 1 (33.3) | 0 (0.0) | | 3 (60.0) | 8 (61.5) | | | 15 (45.5) |  |
| **Type of Fundoplication** | | |  |  | |  |  |  |  | |  |  | | |  |  |
| Nissen | 11 (36.7) | 8 (29.6) | 0.467* | 12 (32.4) | | 7 (35.0) | 0.69* | 2 (66.7) | 3 (100.0) | | 2 (40.0) | 2 (15.4) | | | 10 (30.3) | 0.616* |
| Dor | 2 (6.7) | 5 (18.5) |  | 6 (16.2) | | 1 (5.0) |  | 0 (0.0) | 0 (0.0) | | 0 (0.0) | 2 (15.4) | | | 5 (15.2) |  |
| Toupet | 3 (10.0) | 1 (3.7) |  | 3 (8.1) | | 1 (5.0) |  | 0 (0.0) | 0 (0.0) | | 0 (0.0) | 1 (7.7) | | | 3 (9.1) |  |
| None | 14 (46.7) | 13 (48.1) |  | 16 (43.2) | | 11 (55.0) |  | 1 (33.3) | 0 (0.0) | | 3 (60.0) | 8 (61.5) | | | 15 (45.5) |  |
| **Gastropexy** |  |  |  |  | |  |  |  |  | |  |  | | |  |  |
| Yes | 1 (3.3) | 0 (0.0) | 1* | 1 (2.7) | | 0 (0.0) | 1* | 0 (0.0) | 0 (0.0) | | 0 (0.0) | 0 (0.0) | | | 1 (3.0) | 1* |
| No | 29 (96.7) | 27 (100.0) |  | 36 (97.3) | | 20 (100.0) |  | 3 (100.0) | 3 (100.0) | | 5 (100.0) | 13 (100.0) | | | 32 (97.0) |  |
| **30-day Post-Operative Complications (Clavien-Dindo - C - Classification)** | | | | | | | |  |  | |  |  | | |  |  |
| C0 | 28 (93.3) | 27 (100.0) | 0.492* | 35 (94.6) | | 20 (100.0) | 0.536* | 3 (100.0) | 3 (100.0) | | 5 (100.0) | 13 (100.0) | | | 31 (93.9) | 1* |
| C1-C5 | 2 (6.7) | 0 (0.0) |  | 2 (5.4) | | 0 (0.0) |  | 0 (0.0) | 0 (0.0) | | 0 (0.0) | 0 (0.0) | | | 2 (6.1) |  |
| **30-day Post-Operative Re-admission** | | | |  | |  |  |  |  | |  |  | | |  |  |
| Yes | 28 (93.3) | 27 (100.0) | 1* | 35 (94.6) | | 20 (100.0) | 1* | 3 (100.0) | 3 (100.0) | | 5 (100.0) | 13 (100.0) | | | 31 (93.9) | 1* |
| No | 0 (0.0) | 0 (0.0) |  | 0 (0.0) | | 0 (0.0) |  | 0 (0.0) | 0 (0.0) | | 0 (0.0) | 0 (0.0) | | | 0 (0.0) |  |
| **Recurrence** |  |  |  |  | |  |  |  |  | |  |  | | |  |  |
| Yes - symptoms | 0 (0.0) | 4 (14.8) | 0.347* | 0 (0.0) | | 4 (20.0) | **0.01*** | 1 (33.3) | 1 (33.3) | | 1 (20.0) | 1 (7.7) | | | 0 (0.0) | **0.038*** |
| Yes - no symptoms (radiological recurrence) | 0 (0.0) | 1 (3.7) |  | 0 (0.0) | | 1 (5.0) |  | 0 (0.0) | 0 (0.0) | | 1 (20.0) | 0 (0.0) | | | 0 (0.0) |  |
| No | 7 (23.3) | 10 (37.0) |  | 12 (32.4) | | 5 (25.0) |  | 0 (0.0) | 1 (33.3) | | 2 (40.0) | 5 (38.5) | | | 9 (27.3) |  |
| Missing | 23 (76.7) | 12 (44.4) |  | 25 (67.6) | | 10 (50.0) |  | 2 (66.7) | 1 (33.3) | | 1 (20.0) | 7 (53.8) | | | 24 (72.7) |  |

GERD: gastro-esophageal reflux disease; *Chi-square Test; ^T Test; ^^Kruskal-Wallis test

**Supplementary Table 3. Patients' quality of life, post-operative satisfaction, and clinical surgical variables in type 4 hiatal hernia**

|  | **Demeester Score**  **Below Median Values < 1** | **Demeester Score**  **Above Median Values ≥ 2** | **p value** | **GERD-HRQL Score**  **Below Median Values <5** | **GERD-HRQL Score**  **Above Median Values ≥ 6** | **p value** | **Likert 1** | **Likert 2** | **Likert 3** | **Likert 4** | **Likert 5** | **p value** |
| --- | --- | --- | --- | --- | --- | --- | --- | --- | --- | --- | --- | --- |
|  | **13 patients n (%)** | **10 patients n (%)** |  | **17 patients n (%)** | **6 patients n (%)** |  | **0 patients n (%)** | **2 patients n (%)** | **1 patients n (%)** | **4 patients n (%)** | **16 patients n (%)** |  |
| **Sex** |  |  |  |  |  |  |  |  |  |  |  |  |
| Female | 7 (53.8) | 7 (70.0) | 0.669* | 10 (58.8) | 4 (66.7) | 1* | 0 (0.0) | 1 (50.0) | 1 (100.0) | 3 (75.0) | 9 (56.2) | 0.882* |
| Male | 6 (46.2) | 3 (30.0) |  | 7 (41.2) | 2 (33.3) |  | 0 (0.0) | 1 (50.0) | 0 (0.0) | 1 (25.0) | 7 (43.8) |  |
| **Age (years)** | |  |  |  |  |  |  |  |  |  |  |  |
| Mean (SD) | 68.7 (12.2) | 69.0 (5.7) | 0.956^ | 68.1 (10.9) | 71.0 (5.3) | 0.54^ | -- (--) | 74.4 (5.8) | 66.9 (NA) | 68.1 (8.3) | 68.5 (10.8) | 0.771^^ |
| **Smoking Habits** | | |  |  |  |  |  |  |  |  |  |  |
| Non smoker | 12 (92.3) | 7 (70.0) | 0.281* | 14 (82.4) | 5 (83.3) | 1* | 0 (0.0) | 2 (100.0) | 0 (0.0) | 4 (100.0) | 13 (81.2) | 0.225* |
| Smoker/Past smoker | 1 (7.7) | 3 (30.0) |  | 3 (17.6) | 1 (16.7) |  | 0 (0.0) | 0 (0.0) | 1 (100.0) | 0 (0.0) | 3 (18.8) |  |
| **Previous Abdominal Surgery** | | | |  |  |  |  |  |  |  |  |  |
| Yes | 6 (46.2) | 7 (70.0) | 0.402* | 8 (47.1) | 5 (83.3) | 0.179* | 0 (0.0) | 2 (100.0) | 0 (0.0) | 2 (50.0) | 9 (56.2) | 0.559* |
| No | 7 (53.8) | 3 (30.0) |  | 9 (52.9) | 1 (16.7) |  | 0 (0.0) | 0 (0.0) | 1 (100.0) | 2 (50.0) | 7 (43.8) |  |
| **BMI** |  |  |  |  |  |  |  |  |  |  |  |  |
| Mean (SD) | 24.1 (3.3) | 27.0 (4.9) | 0.102^ | 24.0 (3.1) | 29.0 (5.1) | **0.01**^ | -- (--) | 28.1 (3.8) | 31.9 (NA) | 28.6 (5.7) | 23.8 (3.1) | 0.06226^^ |
| **ASA Score** |  |  |  |  |  |  |  |  |  |  |  |  |
| ASA 1 | 1 (7.7) | 0 (0.0) | 0.788* | 1 (5.9) | 0 (0.0) | 0.694* | 0 (0.0) | 0 (0.0) | 0 (0.0) | 0 (0.0) | 1 (6.2) | 0.206* |
| ASA 2 | 10 (76.9) | 7 (70.0) |  | 13 (76.5) | 4 (66.7) |  | 0 (0.0) | 0 (0.0) | 1 (100.0) | 4 (100.0) | 12 (75.0) |  |
| ASA 3 | 2 (15.4) | 3 (30.0) |  | 3 (17.6) | 2 (33.3) |  | 0 (0.0) | 2 (100.0) | 0 (0.0) | 0 (0.0) | 3 (18.8) |  |
| ASA 4 | 0 (0.0) | 0 (0.0) |  | 0 (0.0) | 0 (0.0) |  | 0 (0.0) | 0 (0.0) | 0 (0.0) | 0 (0.0) | 0 (0.0) |  |
| **Associated Diagnosis** | | | |  |  |  |  |  |  |  |  |  |
| Yes (GERD) | 6 (46.2) | 7 (70.0) | 0.402* | 9 (52.9) | 4 (66.7) | 0.66* | 0 (0.0) | 2 (100.0) | 1 (100.0) | 1 (25.0) | 9 (56.2) | 0.356* |
| No | 7 (53.8) | 3 (30.0) |  | 8 (47.1) | 2 (33.3) |  | 0 (0.0) | 0 (0.0) | 0 (0.0) | 3 (75.0) | 7 (43.8) |  |
| **Surgical Approach** | | |  |  |  |  |  |  |  |  |  |  |
| Robotic Assisted | |  |  |  |  |  |  |  |  |  |  |  |
| Yes | 8 (61.5) | 10 (100.0) | **0.046*** | 13 (76.5) | 5 (83.3) | 1* | 0 (0.0) | 2 (100.0) | 1 (100.0) | 3 (75.0) | 12 (75.0) | 1* |
| No | 5 (38.5) | 0 (0.0) |  | 4 (23.5) | 1 (16.7) |  | 0 (0.0) | 0 (0.0) | 0 (0.0) | 1 (25.0) | 4 (25.0) |  |
| Laparoscopic | |  |  |  |  |  |  |  |  |  |  |  |
| Yes | 5 (38.5) | 0 (0.0) | **0.046*** | 4 (23.5) | 1 (16.7) | 1* | 0 (0.0) | 0 (0.0) | 0 (0.0) | 1 (25.0) | 4 (25.0) | 1* |
| No | 8 (61.5) | 10 (100.0) |  | 13 (76.5) | 5 (83.3) |  | 0 (0.0) | 2 (100.0) | 1 (100.0) | 3 (75.0) | 12 (75.0) |  |
| Converted | |  |  |  |  |  |  |  |  |  |  |  |
| Yes | 0 (0.0) | 2 (20.0) | 0.178 | 1 (5.9) | 1 (16.7) | 0.462 | 0 (0.0) | 1 (50.0) | 0 (0.0) | 1 (25.0) | 0 (0.0) | 0.083 |
| No | 13 (100.0) | 8 (80.0) |  | 16 (94.1) | 5 (83.3) |  | 0 (0.0) | 1 (50.0) | 1 (100.0) | 3 (75.0) | 16 (100.0) |  |
| **Type of Mesh** |  |  |  |  |  |  |  |  |  |  |  |  |
| BIO-A | 7 (53.8) | 6 (60.0) | 1* | 10 (58.8) | 3 (50.0) | 0.802* | 0 (0.0) | 0 (0.0) | 1 (100.0) | 1 (25.0) | 11 (68.8) | 0.101* |
| Phasix | 1 (7.7) | 1 (10.0) |  | 1 (5.9) | 1 (16.7) |  | 0 (0.0) | 1 (50.0) | 0 (0.0) | 0 (0.0) | 1 (6.2) |  |
| None | 5 (38.5) | 3 (30.0) |  | 6 (35.3) | 2 (33.3) |  | 0 (0.0) | 1 (50.0) | 0 (0.0) | 3 (75.0) | 4 (25.0) |  |
| **Fundoplication** | |  |  |  |  |  |  |  |  |  |  |  |
| Yes | 4 (30.8) | 5 (50.0) | 0.417* | 8 (47.1) | 1 (16.7) | 0.34* | 0 (0.0) | 0 (0.0) | 0 (0.0) | 1 (25.0) | 8 (50.0) | 0.544* |
| No | 9 (69.2) | 5 (50.0) |  | 9 (52.9) | 5 (83.3) |  | 0 (0.0) | 2 (100.0) | 1 (100.0) | 3 (75.0) | 8 (50.0) |  |
| **Type of Fundoplication** | | |  |  |  |  |  |  |  |  |  |  |
| Nissen | 4 (30.8) | 2 (20.0) | 0.126* | 6 (35.3) | 0 (0.0) | 0.254* | 0 (0.0) | 0 (0.0) | 0 (0.0) | 1 (25.0) | 5 (31.2) | 0.953* |
| Dor | 0 (0.0) | 3 (30.0) |  | 2 (11.8) | 1 (16.7) |  | 0 (0.0) | 0 (0.0) | 0 (0.0) | 0 (0.0) | 3 (18.8) |  |
| Toupet | 0 (0.0) | 0 (0.0) |  | 0 (0.0) | 0 (0.0) |  | 0 (0.0) | 0 (0.0) | 0 (0.0) | 0 (0.0) | 0 (0.0) |  |
| None | 9 (69.2) | 5 (50.0) |  | 9 (52.9) | 5 (83.3) |  | 0 (0.0) | 2 (100.0) | 1 (100.0) | 3 (75.0) | 8 (50.0) |  |
| **Gastropexy** |  |  |  |  |  |  |  |  |  |  |  |  |
| Yes | 3 (23.1) | 1 (10.0) | 0.604* | 3 (17.6) | 1 (16.7) | 1* | 0 (0.0) | 0 (0.0) | 0 (0.0) | 2 (50.0) | 2 (12.5) | 0.307* |
| No | 10 (76.9) | 9 (90.0) |  | 14 (82.4) | 5 (83.3) |  | 0 (0.0) | 2 (100.0) | 1 (100.0) | 2 (50.0) | 14 (87.5) |  |
| **30-day Post-Operative Complications (Clavien-Dindo - C - Classification)** | | | | | | | |  |  |  |  |  |
| C0 | 3 (23.1) | 3 (30.0) | 1* | 4 (23.5) | 2 (33.3) | 0.632* | 0 (0.0) | 0 (0.0) | 0 (0.0) | 4 (100.0) | 2 (12.5) | **0.003*** |
| C1-C5 | 10 (76.9) | 7 (70.0) |  | 13 (76.5) | 4 (66.7) |  | 0 (0.0) | 2 (100.0) | 1 (100.0) | 0 (0.0) | 14 (87.5) |  |
| **30-day Post-Operative Re-admission** | | | | | |  |  |  |  |  |  |  |
| Yes | 1 (7.7) | 0 (0.0) | 1* | 1 (5.9) | 0 (0.0) | 1* | 0 (0.0) | 0 (0.0) | 0 (0.0) | 0 (0.0) | 1 (6.2) | 1* |
| No | 12 (92.3) | 10 (100.0) |  | 16 (94.1) | 6 (100.0) |  | 0 (0.0) | 2 (100.0) | 1 (100.0) | 4 (100.0) | 15 (93.8) |  |
| **Recurrence** |  |  |  |  |  |  |  |  |  |  |  |  |
| Yes - symptoms | 0 (0.0) | 1 (10.0) | 0.182* | 0 (0.0) | 1 (16.7) | 0.618* | 0 (0.0) | 1 (50.0) | 0 (0.0) | 0 (0.0) | 0 (0.0) | 0.2* |
| Yes - no symptoms (radiological recurrence) | 0 (0.0) | 1 (10.0) |  | 1 (5.9) | 0 (0.0) |  | 0 (0.0) | 0 (0.0) | 0 (0.0) | 0 (0.0) | 1 (6.2) |  |
| No | 6 (46.2) | 3 (30.0) |  | 6 (35.3) | 3 (50.0) |  | 0 (0.0) | 0 (0.0) | 0 (0.0) | 2 (50.0) | 7 (43.8) |  |
| Missing | 7 (53.8) | 5 (50.0) |  | 10 (58.8) | 2 (33.3) |  | 0 (0.0) | 1 (50.0) | 1 (100.0) | 2 (50.0) | 8 (50.0) |  |

GERD: gastro-esophageal reflux disease; *Chi-square Test; ^T Test; ^^Kruskal-Wallis test

**Supplementary Table 4. Clinical and surgical variables according to surgical approach (intention-to-treat) in type 3 and type 4 hiatal hernias**

|  | **Laparoscopy**  **19 patients**  **n (%)** | **Robotic Assisted**  **61 patients**  **n (%)** | **p value** |
| --- | --- | --- | --- |
| **Sex** |  |  |  |
| Female | 12 (63.2) | 44 (72.1) | 0.568* |
| Male | 7 (36.8) | 17 (27.9) |  |
| **Age (years)** |  |  |  |
| Mean (SD) | 64.0 (13.1) | 67.6 (9.3) | 0.197^ |
| **BMI** |  |  |  |
| Mean (SD) | 25.9 (3.2) | 26.0 (3.8) | 0.877^ |
| **Previous Abdominal Surgery** |  |  |  |
| Yes | 8 (42.1) | 38 (62.3) | 0.183* |
| No | 11 (57.9) | 23 (37.7) |  |
| **ASA Score** |  |  |  |
| ASA 1 | 3 (15.8) | 3 (4.9) | 0.098* |
| ASA 2 | 15 (78.9) | 45 (73.8) |  |
| ASA 3 | 1 (5.3) | 13 (21.3) |  |
| ASA 4 | 0 (0.0) | 0 (0.0) |  |
| **Hiatal Hernia Type** | 0.5 (0.7) | 1.2 (1.2) |  |
| Type 3 | 14 (73.7) | 43 (70.5) | 1* |
| Type 4 | 5 (26.3) | 18 (29.5) |  |
| **Converted Surgery** |  |  |  |
| Yes | 1 (5.3) | 4 (6.6) | 1* |
| No | 18 (94.7) | 57 (93.4) |  |
| **Operative Time (minutes)** |  |  |  |
| Mean (SD) | 18.2 (17.7) | 34.0 (13.5) | **<0.001**^ |
| **Mesh Use** |  |  |  |
| Yes | 7 (36.8) | 37 (60.7) | 0.112* |
| No | 12 (63.2) | 24 (39.3) |  |
| **Fundoplication** |  |  |  |
| Yes | 12 (63.2) | 27 (44.3) | 0.192* |
| No | 7 (36.8) | 34 (55.7) |  |
| **Type of Fundoplication** |  |  |  |
| Nissen | 10 (52.6) | 15 (24.6) | 0.127* |
| Dor | 1 (5.3) | 9 (14.8) |  |
| Toupet | 1 (5.3) | 3 (4.9) |  |
| None | 7 (36.8) | 34 (55.7) |  |
| **Gastropexy** |  |  |  |
| Yes | 0 (0.0) | 5 (8.2) | 0.332* |
| No | 19 (100.0) | 56 (91.8) |  |
| **30-day Post-Operative Complications (Clavien-Dindo - C - Classification)** | | | |
| C0 | 17 (89.5) | 55 (90.2) | 1* |
| C1-C5 | 2 (10.5) | 6 (9.8) |  |
| **Post-operative Length of Stay (days)** | |  |  |
| Mean (SD) | 3.1 (0.8) | 3.6 (4.3) | 0.64^ |
| **30-day Post-Operative Re-admission** | | |  |
| Yes | 1 (5.3) | 0 (0.0) | 0.237* |
| No | 18 (94.7) | 61 (100.0) |  |
| **Recurrence** |  |  |  |
| Yes - symptoms | 2 (10.5) | 3 (4.9) | 0.161* |
| Yes - no symptoms (radiological recurrence) | 1 (5.3) | 1 (1.6) |  |
| No | 4 (21.1) | 22 (36.1) |  |
| **Demeester Score Overall** |  |  |  |
| Median (IQR1-IQR3) | 1.0 (0.0-2.0) | 1.0 (0.0-2.0) | 0.6026^^ |
| **GERD Health-Related Quality of Life (GERD-HRQL) Score Overall** | | |  |
| Median (IQR1-IQR3) | 2.5 (0.0-8.5) | 3.0 (0.0-7.0) | 0.8423^^ |
| **Likert Scale Score** |  |  |  |
| 1 | 1 (5.3) | 2 (3.3) | 0.938* |
| 2 | 1 (5.3) | 4 (6.6) |  |
| 3 | 1 (5.3) | 5 (8.2) |  |
| 4 | 5 (26.3) | 12 (19.7) |  |
| 5 | 11 (57.9) | 38 (62.3) |  |

GERD: gastro-esophageal reflux disease; *Chi-square Test; ^ T Test; ^^ Mann-Whitney U test

**Supplementary Table 5. Post-operative complications and quality of life according to mesh use and fundoplication in type 3 and type 4 hiatal hernias.**

|  | **Mesh Use No**  **36 patients**  **n (%)** | **Mesh Use Yes**  **44 patients**  **n (%)** | **p value** | **Fundoplication No**  **41 patients**  **n (%)** | **Fundoplication No**  **39 patients**  **n (%)** | **p value** |
| --- | --- | --- | --- | --- | --- | --- |
| **Hiatal Hernia Type** | |  |  |  |  |  |
| Type 3 | 28 (77.8) | 29 (65.9) | 0.322* | 27 (65.9) | 30 (76.9) | 0.328* |
| Type 4 | 8 (22.2) | 15 (34.1) |  | 14 (34.1) | 9 (23.1) |  |
| **Operative Time (minutes)** | |  |  |  |  |  |
| Mean (SD) | 29.2 (18.8) | 31.1 (13.4) | 0.595^ | 30.6 (15.5) | 29.9 (16.7) | 0.849^ |
| **30-day Post-Operative Complications (Clavien-Dindo - C - Classification)** | | | |  |  |  |
| C0 | 3 (8.3) | 5 (11.4) | 0.724* | 5 (12.2) | 3 (7.7) | 0.713* |
| C1-C5 | 33 (91.7) | 39 (88.6) |  | 36 (87.8) | 36 (92.3) |  |
| **30-day Post-Operative Re-admission** | | | |  |  |  |
| Yes | 0 (0.0) | 1 (2.3) | 1* | 1 (2.4) | 0 (0.0) | 1* |
| No | 36 (100.0) | 43 (97.7) |  | 40 (97.6) | 39 (100.0) |  |
| **Recurrence** |  |  |  |  |  |  |
| Yes - symptoms | 2 (5.6) | 3 (6.8) | 1* | 3 (7.3) | 2 (5.1) | 0.259* |
| Yes - no symptoms (radiological recurrence) | 1 (2.8) | 1 (2.3) |  | 0 (0.0) | 2 (5.1) |  |
| No | 8 (22.2) | 18 (40.9) |  | 17 (41.5) | 9 (23.1) |  |
| Missing | 25 (69.4) | 22 (50.0) |  | 21 (51.2) | 26 (66.7) |  |
| **Demeester Score Overall** | | |  |  |  |  |
| Median (IQR1-IQR3) | 1.0 (0.0-2.0) | 2.0 (0.0-2.0) | 0.621^^ | 1.0 (0.0-3.0) | 1.0 (0.0-2.0) | 0.9802^^ |
| **GERD Health-Related Quality of Life (GERD-HRQL) Score Overall** | | | |  |  |  |
| Median (IQR1-IQR3) | 3.0 (0.8-8.0) | 3.5 (0.0-7.3) | 0.9766^^ | 4.0 (0.0-8.0) | 3.0 (0.5-6.5) | 0.5235^^ |
| **Likert Scale Score** | |  |  |  |  |  |
| 1 | 2 (5.6) | 1 (2.3) | 0.822* | 1 (2.4) | 2 (5.1) | 0.627* |
| 2 | 3 (8.3) | 2 (4.5) |  | 2 (4.9) | 3 (7.7) |  |
| 3 | 3 (8.3) | 3 (6.8) |  | 4 (9.8) | 2 (5.1) |  |
| 4 | 8 (22.2) | 9 (20.5) |  | 11 (26.8) | 6 (15.4) |  |
| 5 | 20 (55.6) | 29 (65.9) |  | 23 (56.1) | 26 (66.7) |  |

GERD: gastro-esophageal reflux disease; *Chi-square Test; ^ T Test; ^^ Mann-Whitney U test

**Supplementary Table 6: Type of Fundoplication and Quality of Life**

|  | **Toupet and Dor Fundoplication**  **(15 patients)** | **Nissen Fundoplication**  **(29 patients)** | **p value** |
| --- | --- | --- | --- |
| **Demeester Score Overall** |  |  |  |
| Median (IQR) | 2 (1-2) | 1 (0-2) | 0.3669 |
| **GERD Health-Related Quality of Life (GERD-HRQL) Score Overall** |  |  |  |
| Median (IQR) | 3 (2-4) | 2 (0-8) | 0.5436 |
| **Likert Scale Score** |  |  |  |
| Median (IQR) | 5 (5-5) | 5 (3-5) | 0.1369 |
